# Supplementary material for: Persistence of Long COVID Symptoms Two Years After SARS-CoV-2 Infection: A Prospective Longitudinal Cohort Study
Source: Viruses. 2024 Dec 20;16(12):1955. doi: 10.3390/v16121955 (PMC11680455; doi:10.3390/v16121955)
Supplement: Supplementary file 1 [file viruses-16-01955-s001.zip › viruses-3343227-supplementary.pdf]

Title: **Persistence of Long COVID symptoms two years after SARS-CoV-2 infection: a longitudinal study**

## Supplements


| | | page |
|---|---|---|
| **Table S1** | Questionnaire for data collection | 2 |
| **Table S2** | Long COVID Questionnaire | 4 |
| **Table S3** | Distribution of long COVID symptoms according to patterns | 7 |
| **Table S4** | Demographic differences between subject who answered the questionnaire compared to those who didn't answer the questionnaire | 7 |
| **Table S5** | Univariate analysis of RBD IgG and Neutralizing Antibody titer between the long COVID groups | 8 |
| **Figure S1A** | NfL Concentration (pg/ml) of subjects reporting persistent cognitive-emotional long COVID symptoms compared to subjects without long COVID symptoms by age (mean with 95% CI). | 9 |
| **Figure S1B** | NfL Concentration (pg/ml) of subjects reporting persistent cognitive long COVID symptoms compared to subjects without long COVID symptoms on infection day and 3-4 weeks after infection | 10 |

**Table S1 – Questionnaire for data collection (carried out via telephone between days 20-90 after recruiting day during the Delta surge**)

| | Question | Answer 1 | Answer 2 | Answer 3 | Answer 4 |
|---|---|---|---|---|---|
| 1. | What is your date of birth? | | | | |
| 2. | What is your gender? | Male | Female | | |
| 3. | What is your current height in m? | | | | |
| 4. | What is your current weight in kg? | | | | |
| 5. | What is the exact date you received a positive PCR test for SARS-CoV-2? | | | | |
| 6. | How many vaccines against SARS-CoV-2 have you received? | 0 | 1 | 2 | 3 |
| 7. | What are the dates you were vaccinated? | | | | |
| 8. | Have you been infected with SARS-CoV2 before July 2021? | Yes | No | | |
| 9. | If you were infected before, what is the date of your previous infection? | | | | |
| 10. | Do you have any comorbidities? | 0 | 1 | 2 | 3+ |
| 11. | Do you suffer from systemic hypertension (systolic blood pressure above 140) for which you are pharmaceutically treated? | Yes | No | | |
| 12. | Do you suffer from dyslipidemia (total cholesterol above 200 or LDL cholesterol above 160) for which you are pharmaceutically treated? | Yes | No | | |
| 13. | Do you suffer from an autoimmune disease for which you are pharmaceutically treated? | Yes | No | | |
| 14. | Do you have diabetes (HbA1C>6.5 or fasting blood sugar>126) for which you are pharmaceutically treated? | Yes | No | | |
| 15. | Do you suffer from heart disease for which you are pharmaceutically treated? | Yes | No | | |
| 16. | Do you suffer from lung diseases such as asthma, COPD, and pulmonary fibrosis for which you are pharmaceutically treated? | Yes | No | | |
| 17. | Do you suffer from any coagulation disorder resulting in | Yes | No | | |

| | | | | | |
|---|---|---|---|---|---|
| | hemorrhage or thrombosis for which you are pharmaceutically treated? | | | | |
| 18. | Are you immunosuppressed (organ transplant recipient, currently undergoing biologic therapy/chemotherapy, treated with corticosteroids, underwent a splenectomy, or diagnosed with HIV)? If yes, please state the specific condition | Yes | No | | |
| 19. | Have you ever had a serious allergic reaction (anaphylaxis) that required immediate treatment? | Yes | No | | |
| 20. | Do you have a liver disease as cirrhosis, hepatitis, liver cancer, or a metabolic disorder? | Yes | No | | |
| 21. | Do you have a kidney disease (a creatinine level of >1.2 mg/dL or GFR<60) for which you are pharmaceutically treated? | Yes | No | | |
| 22. | Are you currently pregnant (as confirmed by a beta-HCG blood test and fetal heartbeat detection on ultrasonography)? | Yes | No | | |
| 23. | Did you experience any illness symptoms? | Yes | No | | |
| 24. | Did you experience a fever above 37.5? How many days did it last? | Yes | No | Number of days | |
| 25. | Did you experience fatigue or weakness? | Yes | No | | |
| 26. | Did you experience muscle aches (myalgia)? | Yes | No | | |
| 27. | Did you experience a headache? | Yes | No | | |
| 28. | Did you experience reduced sense of test and smell? | Yes | No | | |
| 29. | Did you experience shortness of breath? | Yes | No | | |
| 30. | Did you experience cough? | Yes | No | | |
| 31. | Did you experience rhinorrhea? | Yes | No | | |
| 32. | Did you experience a sore throat? | Yes | No | | |
| 33. | Did you experience gastrointestinal inconvenience? | Yes | No | | |
| 34. | Did you experience any other symptoms? | Describe | | | |
| 35. | Did you require oxygen? | Yes | No | | |
| 36. | Did you require hospitalization? | Yes | No | | |

| | | | | | |
|---|---|---|---|---|---|
| 37. | Did you require intensive care? | Yes | No | | |
| 38. | During the days you were infected with SARS-CoV-2, did you sleep in a different room/bed? | Yes | No | | |
| 39. | During the days you were infected with SARS-CoV-2, did you eat separately from your family? | Yes | No | | |
| 40. | During the days you were infected with SARS-CoV-2, did you use a separate bathroom from your family? | Yes | No | | |
| 41. | During the days you were infected with SARS-CoV-2, did you use a mask when moving around at home? | Yes | No | | |

(The questionnaire was reviewed and approved by the Institutional review board of the Sheba Medical Center).

**Table S2 – Long COVID Questionnaire**

| | Question | Answer 1 | Answer 2 | Answer 3 | Answer 4 | Answer 5 |
|---|---|---|---|---|---|---|
| 1. | What is your date of birth? | | | | | |
| 2. | What is your gender? | Male | Female | | | |
| 3. | What is your current height in cm? | | | | | |
| 4. | What is your current weight in kg? | | | | | |
| 5. | Are you smoking? | Never | I smoked in the past | Yes | | |
| 6. | When was the last time you had COVID-19 infection? | | | | | |
| | The following questions refer to the COVID-19 disease you suffered from between July – October 2021 (the Delta wave- the time you participated in the study) | | | | | |
| 7. | After the acute COVID-19 infection, did you suffer from persistent symptoms that lasted | I did not suffer at all, I suffered for a week, | I suffered for 2-12 weeks | I suffered for more than 3 months | I still suffer | |

| | | | | | | |
|---|---|---|---|---|---|---|
| | more than two weeks from the beginning of the acute infection? | I suffered for 1-2 weeks | | | | |
| | Only those who answered: "I suffered for more than 3 months", or "I still suffer", continue with the questionnaire: | | | | | |
| 8. | Please mark the symptoms of long COVID you suffered from for more than 3 months: | Yes | No | | | |
| | Fatigue/exhaustion | Yes | No | | | |
| | Numbness/tingling in hand or feet | Yes | No | | | |
| | Concentration difficulties | Yes | No | | | |
| | Memory disturbance/confusion | Yes | No | | | |
| | Feeling of sadness/despondency/depression | Yes | No | | | |
| | Feeling nervous or anxious | Yes | No | | | |
| | Sleeping difficulties | Yes | No | | | |
| | Cough | Yes | No | | | |
| | Headache | Yes | No | | | |
| | Muscle pain | Yes | No | | | |
| | Joint pain | Yes | No | | | |
| | Hair loss | Yes | No | | | |
| | Decreased physical fitness | Yes | No | | | |
| | Chest pain | Yes | No | | | |
| | Palpitation/ accelerated heart rate | Yes | No | | | |
| | Abdominal pain | Yes | No | | | |
| | Nausea/vomiting/diarrhea | Yes | No | | | |
| | Disturbance in the sense of taste and smell | Yes | No | | | |
| | Shortness of breath at rest | Yes | No | | | |
| | Shortness of breath when I get dressed or that prevents leaving the house | Yes | No | | | |

| | | | | | | |
|---|---|---|---|---|---|---|
| | Shortness of breath when that causes you to stop and rest after walking for 100 meters or for several minutes of walking | Yes | No | | | |
| | Shortness of breath when walking fast on plateau or walking uphill | Yes | No | | | |
| 9. | For each symptom you indicated that you suffered from, please indicate how did the symptom interfere with your day-to-day functioning? | I suffered from the symptom but it did not interfere with my daily functioning. | The symptom interfered with daily functioning at a low level. | The symptom interfered with daily functioning moderately | The symptom interfered with daily functioning to a high degree | The symptom interfered with daily functioning to a very high degree |
| 10. | For each symptom you indicated that you suffered from, please indicate how long have you had the symptom? | I suffered from the symptom for less than 3 months after the COVID-19 infection. | I suffered from the symptom for 3-6 months after the COVID-19 infection | I suffered from the symptom for over 6 months after the COVID-19 infection | I still suffer from the symptom. | |
| 11. | Have you returned to your state of health as it was before you got sick with COVID-19? | Yes | No | | | |
| 12. | Have you returned to work/studies/other activities you did before the COVID-19 infection with the same volume of activity? | Yes, I'm fully back | Yes, I'm partially back | No, I didn’t return to the same volume of activity | | |

(The questionnaire was reviewed and approved by the Institutional review board of the Sheba Medical Center).

## Table S3- Distribution of long COVID symptoms according to patterns

| Fatigue | Pain | Cognitive-emotional | Pulmonary | Headache | Cardiac | Taste and smell | Other symptoms N (%) |
|---|---|---|---|---|---|---|---|
| Fatigue/exhaustion; Decreased physical fitness | Joint pain; Muscle pain; Numbness/tingling in hand or feet | Sleeping difficulties; Feeling nervous or anxious; Feeling of sadness/despondency/depression; Memory disturbance/confusion. Concentration difficulties | Shortness of breath at rest or exertion; Cough | Headache | Palpitation/ accelerated heart rate; Chest pain | Disturbance in the sense of taste and smell | Nausea/vomiting/diarrhea; Abdominal pain; Hair loss |

## Table S4- Demographic differences between the subjects who answered the questionnaire (responders) compared to those who did not answer the questionnaire (non- responders).

| | Non- responders- Subjects that did not answer the questionnaire | Responders- Subjects that answered the questionnaire | P value |
|---|---|---|---|
| N | 935 | 323 | |
| Age (mean, SD) | 42.6, 13.3 | 45.3, 14.2 | 0.001 |
| Sex -Female | 501, 53.6% | 187, 57.9% | 0.18 |
| IgG | | | |
| <300 | 666, 71.2% | 237, 73.4% | 0.65 |
| 300-500 | 91, 9.7% | 32, 9.9% | |
| >500 | 178, 19.0% | 54, 16.7% | |
| Neutralizing Abs | | | |
| 0-512 | 304, 77.0% | 689, 77.6% | 0.8 |
| >1024 | 91, 23.0% | 199, 22.4% | |
| Comorbidities | | | |
| No Comorbidities | 635, 77.2% | 236,73.3% | 0.14 |
| One Comorbidity | 149, 18.1% | 60, 18.3% | |
| >2 | 36, 4.4% | 25, 7.8% | |
| Immunosuppressed | 3, 0.4% | 1, 0.4% | |
| BMI | | | |
| <25 | 27, 3.3% | 6, 1.9% | 0.0072 |
| 25-30 | 417, 51.2% | 135, 42.6% | |
| 30-35 | 256, 31.5% | 111, 35.0% | |
| >35 | 114, 14.0% | 65, 20.5% | |
| Severity of COVID-19 infection | | | |
| Asymptomatic-negligible | 256, 31.1% | 83, 25.8% | 0.144 |
| Mild | 371, 45.1% | 149, 46.3% | |
| Moderate-sever | 196, 23.8% | 90, 28.0% | |

**Table S5 – Univariate analysis of RBD IgG and Neutralizing Antibody titer between the long COVID groups**

| | All | Without long COVID symptoms | With long COVID symptoms | With persistent long COVID symptoms | p-value |
|---|---|---|---|---|---|
| **IgG Mean, (SD)** | 857.4, (2254.4) | 920.7, (2407.2) | 726.4, (1924.6) | 739.9 (1937.6) | 0.601 |
| **Neutralizing Ab. Mean (SD)** | 1247.2, (2705.8) | 1304.5, (2784.9) | 985.4 (2413.9) | 1226.1 (2638.1) | 0.984 |
| **IgG - categories** | | | | | |
| **<300** | 301(70.1%) | 195 (69.4%) | 45 (78.9%) | 61 (69.3%) | 0.665 |
| **300-500** | 38 (8.9%) | 24 (8.5%) | 4 (7.0%) | 10 (11.4%) | |
| **>500** | 87 (20.4%) | 62 (22.1%) | 8 (14.1%) | 17 (19.3%) | |
| **Neutralizing Ab.- categories** | | | | | |
| **0-512** | 307 (77.7%) | 205 (78.0%) | 41 (80.4%) | 61 (75.3%) | 0.625 |
| **>1024** | 88 (22.3%) | 58 (22.0%) | 10 (19.6%) | 20 (24.7%) | |

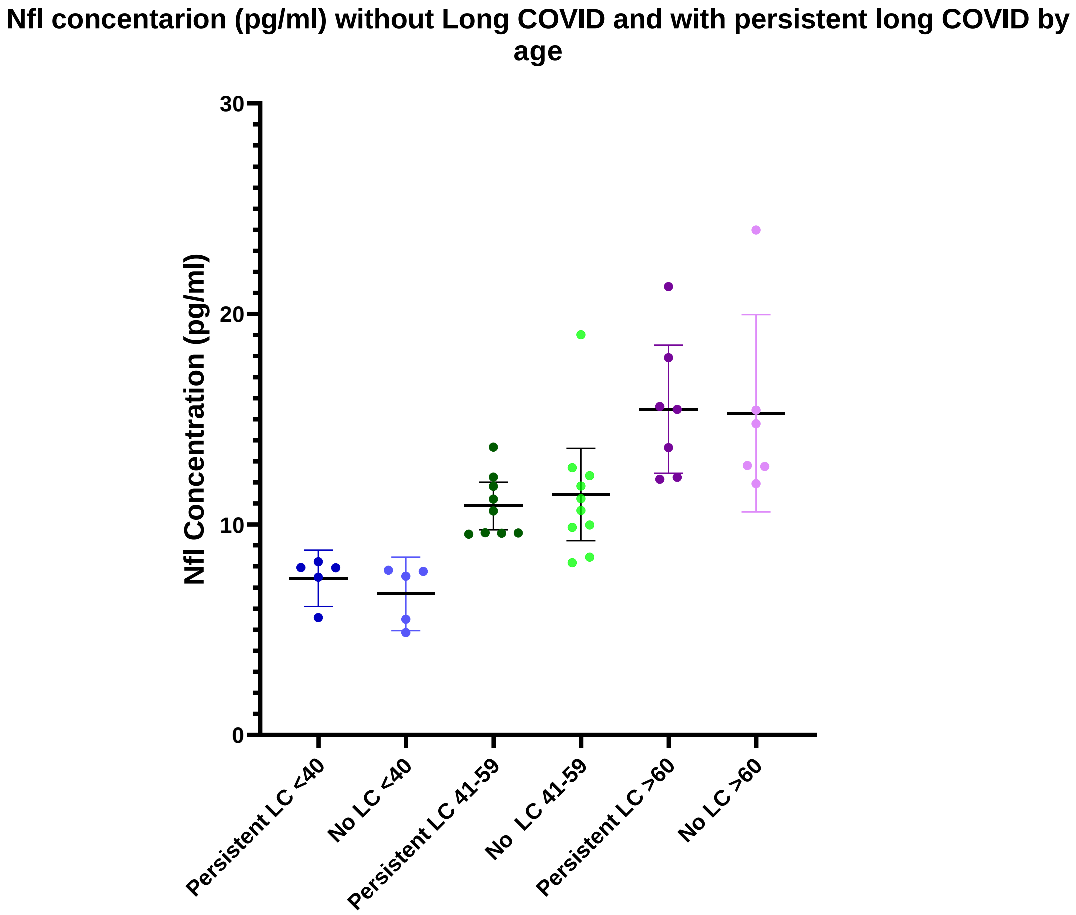


**Figure S1A**- NfL Concentration (pg/ml) of subjects reporting persistent cognitive-emotional long COVID symptoms compared to subjects without long COVID symptoms by age (mean with 95% CI).

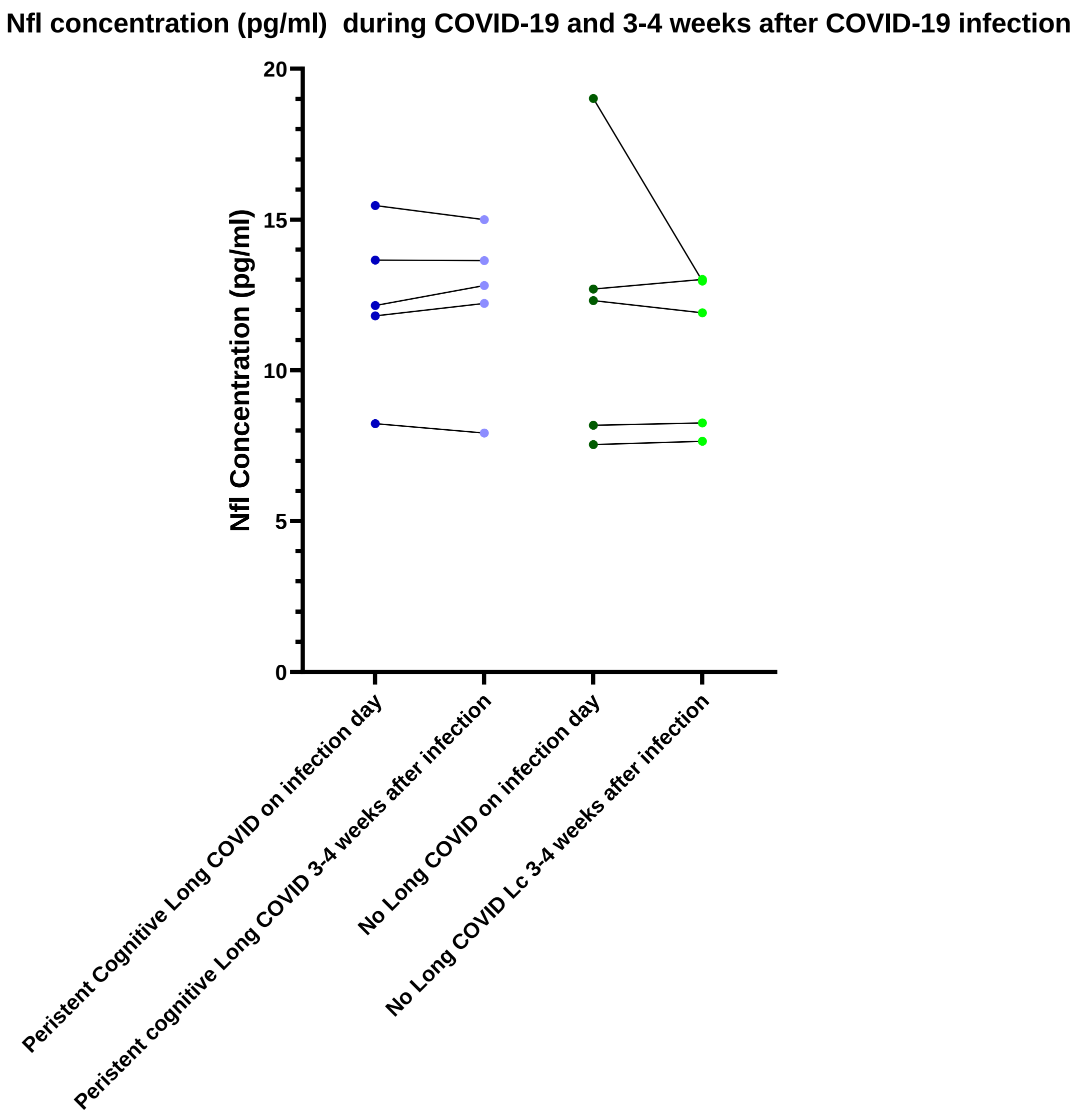


Figure S1B- NfL Concentration (pg/ml) of subjects reporting persistent cognitive long COVID symptoms compared to subjects without long COVID symptoms on infection day and 3-4 weeks after infection.
